# Supplementary material for: Elevated plasma free thiols are associated with early and one-year graft function in renal transplant recipients
Source: PLoS One. 2021 Aug 11;16(8):e0255930. doi: 10.1371/journal.pone.0255930 (PMC8357095; doi:10.1371/journal.pone.0255930)

S1 Fig. The level of plasma free thiols at different time points depending on treatment (Sham-RIC vs. RIC). RIC = remote ischaemic conditioning.


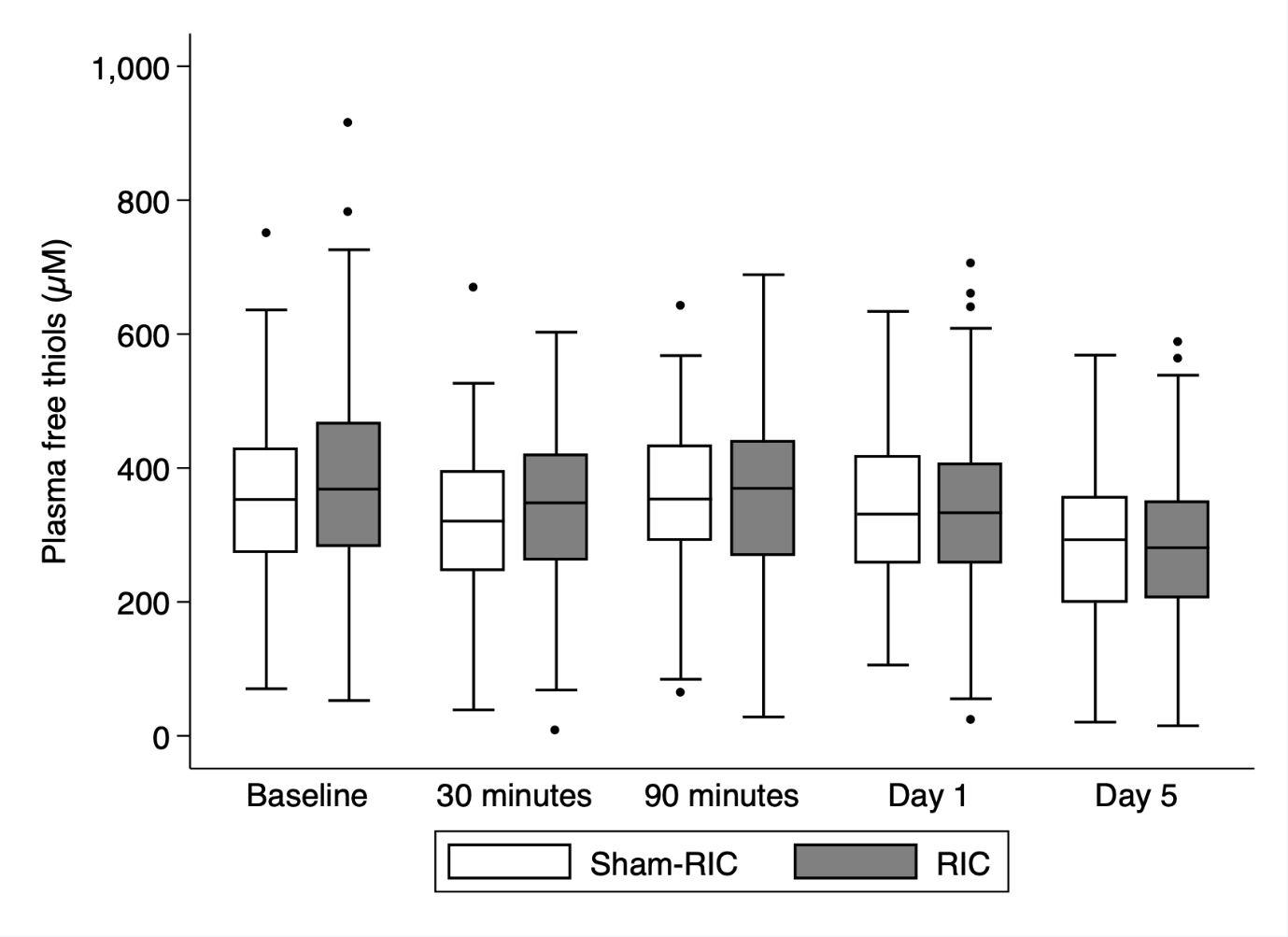

Supplement: S1 Fig — RIC = remote ischaemic conditioning. (DOCX) [file pone.0255930.s001.docx]
